# Psychometric Assessment of the Physicians’ Job Demands and Resources Scale

**DOI:** 10.1177/01632787231195077

**Published:** 2023-08-16

**Authors:** Sérgio Moreira, Sofia Oliveira, Jorge Vala, Rui Costa-Lopes, Alexandra Marques-Pinto

**Affiliations:** 1 Faculdade de Psicologia, 449663 Universidade de Lisboa, CICPSI, Lisboa, Portugal; 2 56060 Instituto de Ciências Sociais da Universidade de Lisboa, Lisboa, Portugal

**Keywords:** burnout, physicians, organizational demands and resources, psychometric testing, factor analysis

## Abstract

Job demands and resources have been consistently associated with the burnout syndrome in physicians, however the literature points to a lack of robust measures to assess these job characteristics across various medical specialties. This study aimed to develop a theoretically and empirically grounded physician-specific job demands and resources self-report measure – the *Physicians’ Job Demands and Resources Scale*. Relevant dimensions of physicians’ job demands and resources were identified, corresponding measurement items were generated and pre-tested, and the factor structure of the resulting 44 items was tested with a sample of 9,176 Portuguese physicians. The results of EFAs and CFAs with two random split samples provided consistent evidence of a nine-factor structure with 38 of the 44 items. Importantly, the nine-factor structure is consistent with the dimensions identified in the literature. The paper discusses the theoretical and practical impacts of the scale.

## Introduction

Physicians’ burnout has been studied extensively over the past four decades with a view to preventing and mitigating its purportedly high prevalence and impacts on individual physicians and their family’s well-being, patients, health care organizations and society at large (Marques-Pinto et al., 2021; Williams et al., 2020). Burnout is a recognized public health problem (World Health Organization, 2019) that has been conceptualized as a triadic syndrome (emotional exhaustion, depersonalization, and low sense of personal accomplishment at work; Maslach, 1976) and negatively linked to job performance and individual well-being (e.g., Panagioti et al., 2017; Williams et al., 2020). Although job demands and resources have been systematically associated with the burnout syndrome, none of the reviewed studies on physician burnout presented a robust measure to assess these variables across the various medical specialties. Additionally, they did not depict and operationalize the specific job demands and resources relevant to the characterization of medical activity. This paper contributes to filling this gap by proposing, testing, and providing a measurement instrument of physicians’ job demands and resources.

### Job Demands and Resources as Determinants of Burnout

From a psychosocial perspective, burnout is an occupational phenomenon that is driven by chronic distress due to unfavorable working conditions that are not successfully managed (Schaufeli et al., 2009; World Health Organization, 2019). The Job Demands-Resources (JD-R) model (e.g., Bakker & Demerouti, 2017; Demerouti et al., 2001; Schaufeli & Taris, 2014) emerges as one of the leading models to help explain and conceptualize how job and personal characteristics are related to burnout development (Marques-Pinto et al., 2021). In accordance with the JD-R model, within any one job it is possible to distinguish between *job demands* and *job resources* (Demerouti et al., 2001). The former are described as negatively valued physical, psychological, social or organizational job characteristics which, due to the requirement of sustained physical and/or psychological effort, are the main predictors of professional impairment, ill-health and burnout (Schaufeli & Bakker, 2004; Schaufeli & Taris, 2014). Job demands can be quantitative (e.g., workload) or qualitative (i.e., emotional) in nature and prolonged exposure to them has been primarily linked to the increase of emotional exhaustion (Schaufeli & Taris, 2014), although specific job demands appear to also predict increased depersonalization (Aronsson et al., 2017; Houkes et al., 2008). Job demands can also be distinguished from demands that are challenging (e.g., time pressure, responsibility) or a hindrance (e.g., role conflict, role ambiguity) (Crawford et al., 2010). On the other hand, job resources include the positively valued physical, psychological, social and organizational job characteristics that contribute to reducing perceived job demands and their costs, to facilitating the achievement of work-related goals, and/or to supporting growth, learning and personal development (Schaufeli & Bakker, 2004; Schaufeli & Taris, 2014). The absence of adequate job resources (e.g., social support, autonomy, feedback) has been mainly linked to depersonalization and loss of personal accomplishment (Houkes et al., 2008; Schaufeli & Taris, 2014).

The JD-R model has a heuristic nature and is highly flexible across work settings since it considers that any perceived job demand and resource can affect employees’ occupational health, well-being and engagement (Schaufeli & Taris, 2014). More specifically, job demands are associated with a depletion of energy, while job resources can, by definition, promote motivation and engagement towards work, thus representing more than an absence of job demands (Schaufeli & Taris, 2014). Despite this conceptual distinction, in practice, differentiation between these two types of job characteristics is not so clearly defined. Across different studies, the same job characteristic can be found identified as a job resource (e.g., “role clarity” in Bakker et al., 2007; “autonomy” in Lee et al., 2013) or a job demand (e.g., “role ambiguity” in Crawford et al., 2010; “lack of autonomy” in Maslach et al., 2001) depending on the operationalization of the items (e.g., positive or negative; present or absent).

### The Importance of the Specificity of Burnout Determinants

Despite the reciprocal interaction between job and personal characteristics (Bakker & de Vries, 2021; Bakker & Demerouti, 2017; Guthier et al., 2020), job demands and resources seem to be the main predictors of the development of physicians’ burnout symptoms (Marques-Pinto et al., 2021) and organization-targeted interventions addressing the work environment and organizational culture seem to be the most effective in reducing physician burnout (Panagioti et al., 2017). Indeed, although more effective, organization-targeted interventions are also more heterogenous and entail higher costs (Panagioti et al., 2017), thus pointing to the need to understand the role of specific job characteristics and tailor interventions to the specific needs of different professional groups.

The literature maintains that occupation-specific burnout profiles (Schaufeli & Buunk, 2003) interfere with and shape the role and effect of particular job characteristics, e.g., physicians are particularly susceptible to work-family interference (Montgomery et al., 2006), and when compared to blue collar workers, the combination of high demands and unavailability of resources is more psychologically detrimental for physicians (Hu et al., 2011). However, research to date has mainly addressed and described the development and prevalence of physician burnout symptoms (e.g., Rotenstein et al., 2018) and their consequences (e.g., Williams et al., 2020), while less attention has been given to their determinants (Chênevert et al., 2021), thus making it difficult to grasp the precise nature (i.e., demand or resource) and effects of the various relevant job characteristics (Lee et al., 2021).

### Fragilities in the Research of Burnout Determinants

Findings on the determinants of physician burnout are inconsistent across the literature (Houkes et al., 2008). Moreover, few studies have specifically tested the JD-R model with physicians compared to other professional groups (Schaufeli & Taris, 2014), and a consensus regarding the instruments to assess physicians’ perception of job demands and resources is also lacking (Aronsson et al., 2017). Although the research has emphasized the need to resort to valid and robust measures that assess the organizational variables that can predict physician burnout in order to acknowledge the role and contribution of different job characteristics (Panagioti et al., 2017; Rotenstein et al., 2018), most studies have used general measures, which do not take into consideration profession specific-characteristics (Zeng et al., 2021) and also various scales and/or isolated items to ensure coverage of all the variables under assessment (e.g., Bakker et al., 2007; Schaufeli & Bakker, 2004).

When considering the process underlying the selection of the job demands and resources in the various studies, some are often reported as having been selected on the basis of previous works (e.g., social support from colleagues, workload, job control) while others on the basis of existing general theories that can be related to burnout (e.g., autonomy, participation in decision-making processes) (e.g., Houkes et al., 2008; Lee et al., 2013; Vandenbroeck et al., 2017). However, there appears to be no consensus as to the job demands and/or resources that predict physician burnout. Given their relevance, they should be assessed when evaluating contexts and designing interventions. Also, to the best of our knowledge, no theoretically grounded and validated measure is available to assess physicians' specific job demands and job resources. Therefore, it is necessary to take a step back and ascertain physicians’ perceptions of their work environments to clarify and identify potential unknown job demands and/or resources which predict physician burnout (Williams et al., 2020), and then develop a robust and integrated measure that will enable the assessment of physicians’ perceived job demands and resources.

### The Current Study

The literature review revealed that studies on physician burnout did not present a robust measure to assess these variables across the various medical specialties. Additionally, they did not depict and operationalize the specific job demands and resources relevant to the characterization of medical activity. Hence, aiming to develop a theoretically and empirically grounded physician-specific job demands and resources self-report measure, this study presents the *Physicians’ Job Demands and Resources Scale* and tests its factor structure. This scale covers the relevant dimensions of this professional group’s job characteristics based on a literature review of the main job demands and resources and through assessment needs validation with physicians.

## Method

### Sample

A total of 43,983 physicians registered in the *Ordem dos Médicos* [Portuguese Medical Association] (OM) with eligible emails were invited to participate in the study, and 9,176 physicians replied to the invitation (21% response rate). Among the 9,176 study participants, 53% were female; 29.5% were aged up to 35 years, 28.7% between 35 and 55 years, and the remaining 41.9% were over 55 years of age. Furthermore, 35.0% belonged to the regional delegation of the OM in the north, 16.9% to the center and the remaining 48.1% to the south delegation. Regarding the participants' professional practice, they had 12 years of experience on average; the majority worked either in hospitals (64%) or in clinics (25%); 45% were specialists, 24% interns, and 25% consultants; and 58% worked in at least 2 different workplaces. Our sample included physicians from a range of 48 specialties, the most frequent being General Practice (25.8%), Internal Medicine (9.8%), Pediatrics (6.8%), Anesthesiology (5.2%) and General Surgery (4.2%). A full description of the specialties of the participants can be found elsewhere (Vala et al., 2017). The procedure followed to ensure that the distribution of the study sample (e.g., gender, age and regional affiliation to the OM) was the same as that of the population from which it was extracted has been described elsewhere; Marques-Pinto et al., 2021).

### Development of the Physicians’ Job Demands and Resources Scale

#### Item Generation Process

To identify the main dimensions of physicians’ job demands and resources, a literature review was conducted on 1) studies generically covering job demands and resources across different professions (e.g., Schaufeli & Taris, 2014) and 2) studies on physician burnout in which these types of variables had been considered (e.g., Houkes et al., 2008; Lee et al., 2013). On the basis of this literature review, an inductive content analysis approach following Bardin’s guidelines (1977) supported the items’ generation process. Prior studies integrated the *corpus* to be analyzed. The studies were analyzed and representative observable expressions of job demands or job resources (e.g., “long work hours”) were encoded as relevant recording units. Specific job demands and resources dimensions then emerged as categories (e.g., Quantitative demands relative to workload/time pressure). This process led to the identification of seven potentially relevant job demands and resources dimensions for physicians, along with representative observable statements for each dimension. The following dimensions emerged:

- (1) *Physical demands* (with statements such as “lack of comfortable rooms for doctors on call”, “Ergonomics and work-related hazards”; e.g., Al-Dubai & Rampal, 2010; Bakker et al., 2007; Le Blanc & Schaufeli, 2003; Schaufeli & Taris, 2014);
- (2) *Cognitive demands* (with statements such as “amount of knowledge that is not easy to assimilate”, “decision making has become more complicated”; e.g., Le Blanc & Schaufeli, 2003; Schaufeli & Taris, 2014; Schaufeli et al., 2009);
- (3) *Organizational demands* (with statements such as “conflicting demands of the job have to be met”, “paperwork volume”; e.g., Maslach et al., 2001; Schaufeli & Taris, 2014; Schaufeli et al., 2009, 2011);
- (4) *Demands of the relationship with patients* (with statements such as “repeated exposure to suffering”, “uncooperative patients”; e.g., Al-Dubai & Rampal, 2010; Bakker et al., 2000, 2007; Le Blanc & Schaufeli, 2003; Lee et al., 2013; Maslach et al., 2001; Schaufeli & Buunk, 2003; Schaufeli & Taris, 2014; Schaufeli et al., 2011);
- (5) *Demands of relationships in the workplace* (with statements such as “negative aspects of relationships with colleagues”, “unresolved conflict with others on the job”; e.g., Al-Dubai & Rampal, 2010; Le Blanc & Schaufeli, 2003; Lee et al., 2013; Maslach et al., 2001; Schaufeli & Taris, 2014);
- (6) *Quantitative demands relative to workload/time pressure* (with statements such as “long work hours”, “too much work to do in too little time”; e.g., Le Blanc & Schaufeli, 2003; Maslach et al., 2001; Montgomery et al., 2006; Schaufeli & Bakker, 2004; Schaufeli & Buunk, 2003);
- (7) *Job resources* (with statements such as “opportunities for professional development”, “social support from colleagues and social support from supervisor”; e.g., Bakker et al., 2007; Le Blanc & Schaufeli, 2003; Schaufeli & Bakker, 2004; Schaufeli & Taris, 2014).

Although the revised JD-R model integrates personal resources (Schaufeli & Taris, 2014), this dimension was not included in the scale. This option was taken due to the fact that personal resources are less specific (e.g., neuroticism), and robust, valid measures to assess them are available for all professional groups (e.g., Big Five Inventory; John & Srivastava, 1999).

As the recording units underlying the *corpus* of analysis were originally written in English, a translation process to ensure the linguistic equivalence and cultural adequacy of the generated items was performed. The identified representative observable statements were first translated (with a focus on functional and not literal equivalence) from English to Portuguese by two Portuguese independent researchers with a PhD in Psychology, familiarity with the targeted context, and who were fluent in both languages (American Psychological Association, 2020; International Test Commission, 2017). The two translated and adapted versions of the representative observable statements were then compared to their original versions, and the researchers discussed the discrepancies until a consensus was found and a single Portuguese version of the items was created. Lastly, the Portuguese version of the recording units was back-translated by a bilingual researcher, and the original and back-translated versions of the representative observable statements were compared and subsequently deemed similar (American Psychological Association, 2020; International Test Commission, 2017).

An inferential process/interpretation of the data based on the categorized observable statements then led to the generation of a first pool of items (*n* = 38). The items generated from the literature review were validated through three Focus Group interviews with independent physicians from different regional delegations of the OM (North, Center, and South delegations) (International Test Commission, 2017). This pre-test of the survey intended to identify queries about the interpretation and adequacy of the questions. Each Focus Group lasted approximately two hours, in which all participants had space to handle the survey on paper/computer and without interruptions. Then, the physicians were asked to evaluate whether the items were clear and relevant and if each dimension was sufficiently and parsimoniously covered (Escobar-Pérez & Cuervo-Martínez, 2008). As a result of the expert analysis, six items were added to ensure sufficiency of the seven dimensions. This second version was revised by the team from the University of Lisbon, in collaboration with the technical-scientific team of the OM. Thus, the complete version of the scale used in the data collection phase consisted of a total of 44 items covering both demands and resources across the seven dimensions identified in the literature: *Physical demands* (four items); *Cognitive demands* (four items); *Organizational demands* (10 items); *Demands of the relationship with patients* (eight items); *Demands of relationships in the workplace* (five items); *Quantitative demands relative to workload/time pressure* (four items); and *Job resources* (nine items). The items were evaluated on a 11-point *Likert* scale (0 – *Completely disagree* to 10 – *Completely agree*). The full description of the content analysis approach underlying the item generation process is depicted in Table S1 of the Supplementary materials.

### Procedures

#### Data Collection

Data collection was conducted in the context of a broader survey (Marques-Pinto et al., 2021). All the procedures and measures were reviewed and received ethical approval by the Ethics Committee of the OM (more details on the procedures and measures used in the survey can be found in Marques-Pinto et al., 2021).

The survey was disseminated through Qualtrics using a private individual link with a view to minimize multiple responses by the same participant and by participants beyond the study’s universe (Dewitt et al., 2018). The online survey, along with information regarding the study’s purpose, was sent individually by email to each of the 43,983 registered physicians with eligible emails in the OM. Prior to partaking in the study, the participants were asked to sign the informed consent which ensured the confidentiality of the responses and anonymity of the participations and guaranteed voluntary participation and the possibility of dropping out at any time. The data collection process lasted two months. No compensation was offered to the participants.

#### Data Analysis

The descriptive analysis of the items (Table S2, Supplementary materials) revealed that, of the 44 items, four had been answered by over 78% of the participants, and the remaining 40 by over 90%. The items with lower response rates referred to *few resting places* and *moral harassment*, suggesting that these demands might be not as transversal to the physicians’ activity as others such as, *lack of time to perform the necessary task* and *extensive time investment* with 99% completion rates. The analysis of the item statistics for Skewness and Kurtosis suggested the prevalence of asymmetric distributions, particularly among the *Cognitive demands* and the *Quantitative demands relative to workload/time pressure demands* sub-factors. Considering the initial testing of the survey in a focus group, this is likely a sign of the prevalence of the phenomena in the population but it can also result from acquiescence in responses. To control the effect of the distribution asymmetry in the analysis, a database with log transformed variables was computed. This transformation led to mixed results, improving the skew statistics in some items, and worsening on others. Still, all analysis were performed with both datasets - original and transformed - and no differences were found in the results.

##### Measurement Models

In this study the recommendations of other authors were followed to test the factor structure underlying the 44 items used to measure the physicians’ job demands and resources (e.g., Anjos et al., 2019; Cabrera-Nguyen, 2010; Worthington & Whittaker, 2006). To begin, the original database with 9,176 participants was randomly split into two databases using the selected cases/random sample in the SPSS software. The resulting databases each contained 4,588 participants.

One of the databases was then used to run an Exploratory Factor Analysis (EFA) to assess the factor structure and refine the original item pool. The procedure described by Anjos et al. (2019) was adapted for the EFA. The Principal Axis Factoring method was used, and the number of factors was determined considering both the interpretability of the factors and that no factor had an eigenvalue lower than .70. The interpretation of the items in each factor was based on *oblimin* solutions, since demands and resources factors are expected to correlate with each other (Field, 2009; Tabachnick & Fidell, 2007). Items were considered to be held for a particular factor when: communalities were higher than .09, loadings were equal to or higher than .32, and cross-loadings were lower than .32 (Brown, 2006; Field, 2009). Finally, the resulting items in a factor were tested for internal consistency using Cronbach’s alpha. The EFA was conducted using SPSS for Mac OS version 24.0.

The factor structure resulting from the EFA was then tested using a Confirmatory Factor Analysis (CFA) with the other database deriving from the random split. The statistical quality of the models resulting from the CFA was assessed using overall goodness of fit measures with the following guidelines (Brown, 2006): SRMR and RMSEA lower or equal to .08, Comparative Fit Index (CFI) and Tucker-Lewis Index (TLI) higher or equal to .90. Chi-sq as a model fit statistic was reported but, due to its limitations with large samples (Brown, 2006), was not considered to assess the model’s fit. Additionally, in the case of an overall poor fit, measures of the localized areas of strain with the following guidelines were also considered (Brown, 2006): standardized residuals lower or equal to 2.58 and general modification indexes analysis lower or equal to 4. The CFA was conducted using R Studio for mac, version 1.1.453 (R Core Team, 2018), with the function *cfa* from the *lavaan* package (Rosseel, 2012).

##### Conditions Influencing Parametric Estimation

Conditions known to influence the estimation of parameters and adjustment measures in the EFA and CFA (Brown, 2006) were analyzed, namely missing data, symmetry, extreme cases, and items correlations. The sample used to run the EFA showed that the proportion of missing data varied between 1% and 22% of the total sample of 4,588 physicians, with only five items showing proportions above 10%. Considering the study’s large sample size, these five items were maintained in the analysis. Additionally, to account for the potential contribution of the missing data to this factor structure, factor models using both pairwise and listwise deletions were computed and compared.

The results also revealed, with very few exceptions which will be discussed further ahead, a prevalence of negative asymmetric and platykurtic distributions. These distributions indicated that first, the physicians in our sample tended to agree with the inclusion of both the demands and the resources measured, and second, that despite this trend, the responses had considerable variability. It should be noted that, although the statistics for the skewness and kurtosis indicated statistically significant differences from a normal distribution, they did not represent a strong case of departure from normality. First, several authors have noted that when sample sizes are large, significant values arise from even small deviations from normality (Field, 2009; Tabachnick & Fidell, 2007). Second, the visual inspection of P-P plots, boxplots, and histograms revealed good adjustments to the normal distributions and the absence of extreme cases. Third, a transformation of the items using a log10 of the inverted items had little effect on both the skewness and kurtosis statistics and the graphs used in visual inspection. Importantly, the items measuring cognitive demands were an exception to this pattern with more extreme skewness statistics indicating higher levels of acquiescence with these demands, positive and more extreme kurtosis statistics indicating, contrary to the previous items, the presence of leptokurtic distributions, and, finally, the presence of several extreme cases. To account for the potential contribution of these issues to the factor structure of the job resources and demands, factor models using the original items measuring cognitive demands and these same items without the extreme cases were computed and compared. Finally, the analysis of the bivariate correlations of the 44 items revealed that all the correlations ranged between −.56 and .79. This constitutes a good indication that there were no multicollinearity problems in the covariance matrix underlying the factor models (Field, 2009; Tabachnick & Fidell, 2007).

As expected, the sample used to run the CFA showed very similar results to those described above – low proportion of missing data, a prevalence of negative asymmetric and platykurtic distributions with the exception of the items measuring cognitive demands that had more extreme skewness statistics along with leptokurtic distributions, and bivariate correlations of the 44 items between −.57 and .80. Here, to account for the potential contribution of the missing data and data distribution symmetry to the confirmatory measurement model, both maximum likelihood (ML) and robust maximum likelihood (MLR) with direct ML missing data replacement estimations were computed and compared (Brown, 2006).

## Results

### Exploratory Factor Analysis

#### Factor Extraction

The results for the first solution of the EFA suggested nine factors with a total of 51% explained variance. Among the 44 items, five were considered problematic either due to low loadings (Organizational demands_10 loadings < |.25|, Time demands_1 loadings < |.29|) or to cross-loadings (Job resources_6, Organizational demands_5, Organizational demands_3). These items were removed from the analysis and a new EFA was run using the remaining 39 items. The results for this second factor analysis suggested, again, nine factors with a total of 54% of explained variance. One item had low loadings (Organizational demands_2) and was consequently removed from the analysis and a new EFA was run using the remaining 38 items. The results for this third factor model replicated the nine-factor structure with an explained variance of 55% and with no problematic items (see Table 1).

**Table 1.** Results of the Factor Structure of the EFA and Descriptive Statistics of the Extracted Factors.

	1. Resources	2. Patients in pain	3. Cognitive demands	4. Relationship demands	5. Time demands	6. Physical demands	7. Technical demands	8. Difficult patients	9. Lack of autonomy
Factors									
Var. (%)	20.78	9.93	7.16	5.84	3.24	2.91	2.58	1.79	1.28
Alpha/r	.88	.84	.87	.90	.86	.86	.69	.80	.61
M	5.93	6.72	8.75	6.37	7.34	5.31	5.62	6.46	4.82
SD	1.95	2.20	1.31	2.59	2.34	2.46	2.43	1.97	2.28
N	4540	4477	4556	4423	4536	4508	4533	4472	4474
Correlations^1^									
1	—	−.06	.00	−.26	−.23	−.29	−.39	−.19	−.48
2			.20	.22	.16	.11	.15	.54	.18
3				.18	.39	.27	.11	.19	.13
4					.23	.18	.17	.38	.27
5						.28	.28	.25	.36
6							.26	.18	.31
7								.29	.48
8									.31

*Note.* ^1^Correlations are not marked for significance due to the large sample size biasing error estimate and corresponding test statistics.

Importantly, the factor structure was interpretable and in line with the original alignment of the items, with the nine factors corresponding to the seven job demands and resources dimensions identified in the literature review. Eight of the nine items of the *Job resources* dimension formed factor 1 (hereinafter referred to as *Resources* for readability purposes), with the missing item being removed from the analysis due to cross-loadings. The eight items from the *Demands of the relationship with patients’* dimension formed two factors: three items loaded on factor 2 (named *Demands of the relationship with patients in pain*; hereinafter referred to as *Patients in pain*), and the remaining five items on factor 8 (named *Demands of the relationship with difficult patients*; hereinafter referred to as *Difficult patients*). The four items from the *Cognitive demands* dimension formed factor 3 (hereinafter referred to as *Cognitive demands*). The five items of the *Demands of Relationships in the workplace* dimension formed factor 4 (hereinafter referred to as *Relationship demands*). Three of the four items of the *Quantitative demands relative to workload/time pressure* dimension formed factor 5 (hereinafter referred to as *Time demands*), with one item dropping out of the analysis due to low loadings. The four items of the *Physical demands* dimension formed factor 6 (hereinafter referred to as *Physical demands*). Out of the 10 items of the *Organizational demands* dimension, two items were dropped from the analysis due to low loadings, two were dropped due to cross loadings, and the remaining six formed two factors: three items loading on factor 7 (named *Demands due to the lack of adequate technical and diagnostic resources*; hereinafter referred to as *Technical demands*), and three items loading on factor 9 (named *Demands due to the lack of autonomy/participation in decision making*; hereinafter referred to as *Lack of autonomy*). A full report of the results of the third factor model is presented in Table S3 of the Supplementary material.

#### Factor Interpretation

In short, of the 44 original items, 38 were kept in the final factor structure presented. These 38 items loaded on nine different factors in a way that is consistent with the original dimensions identified in the literature review. Importantly, the EFA made it possible to refine the most relevant items and corresponding factors. Here, it should be noted that the items included in the *Organizational demands* dimension resulted in two different factors (*Technical demands* and *Lack of autonomy*) and the items in the *Demands of the relationship with patients* dimension also resulted in two different factors (*Patients in pain* and *Difficult patients*). All the remaining five factors were in line with the original dimensions (*Resources*, *Cognitive demands*, *Relationship demands*, *Time demands*, and *Physical demands*).

The analysis of the data underlying the factor structure revealed that all the factor loadings of the items on the corresponding factors varied (in absolute value) from .34 to .92. Additionally, the levels of explained variance of the nine factors varied between 21% (for the *Resources*) and 1% (for the *Lack of autonomy*), with all the eigenvalues higher than 1.01, and the analysis of the internal consistency of the items composing each of the factors revealing alpha values of between .61 (*Lack of autonomy*) and .90 (*Relationship demands*). It should be noted that given the performance of the *Lack of autonomy* and *Technical demands* factors, they should be interpreted with particular caution. In fact, in view of their similar common-ground dimension, they might even appear on a single factor in future replications.

Finally, for descriptive purposes, the scores for the factors identified in the EFA were computed using coarse scores (i.e., the average rating of each participant on the items composing the factors). The results showed that for seven of the eight factors representing different dimensions of demands, the averages were higher than the midpoint of the scale, indicating a tendency towards more unfavorable perceptions about the workplace. This tendency was particularly marked in the *Cognitive demands* factor, with an average rating of 8.75 (*SD* = 1.31). The only demands dimension that contradicted this result was *Lack of autonomy*, with an average rating of 4.83 (*SD* = 2.28). Finally, for the factor representing *Job resources*, the average rating showed a slight tendency towards more favorable perceptions, with an average rating of 5.93 (*SD* = 1.95). Overall, this descriptive analysis for the factors is consistent with the descriptive analysis preformed for the items in the previous section.

The correlation matrix with the nine factors showed very weak to moderate associations, ranging from .00 to .54 in absolute value. Most correlations were very weak to weak in magnitude (between .11 and .29 in absolute value), with *Resources* yielding a correlation close to zero with *Patients in pain* and *Cognitive demands*, *Cognitive demands* correlating slightly higher than .10 with *Technical demands* and *Lack of autonomy*, and *Patients in pain* also yielding a correlation of .11 with *Physical demands*. Furthermore, some correlations, particularly with the *Lack of autonomy* factor, yielded a moderate magnitude (between .31 and .54). Here, the correlation between *Patients in pain* and *Difficult patients* and between *Lack of autonomy* and both *Resources* and *Technical demands* were among the highest, yielding values above .48. Overall, this matrix suggested that, while some factors might be tapping into similar dimensions (for instance, *Lack of autonomy*/*Technical demands* and *Patients in suffering*/*Difficult patients*), the factors represent distinct, non-redundant dimensions of the demands and resources in the workplace.

#### Alternative Models

The preliminary descriptive analysis reported in the previous section showed that the distribution of missing data varied, with five items showing a higher prevalence of missing data (between 11% and 22%) than the remaining items (all below 8%). Additionally, the descriptive analysis also pointed to a lack of adjustment of the distribution of the data to a normal distribution, particularly among the items representing the *Cognitive demands* factor. To account for the potential contribution of these issues to the factor structure of the scale, two additional EFA were conducted, one using listwise deletions of the cases to deal with the missing data issue, and another using a log_10_ transformation of all the items to deal with the distribution issue of some of the items. The results for these two models replicated the model using the raw data, with two minor exceptions (Table S4, Supplementary materials).

### Confirmatory Factor Analysis

#### Model Fit

In order to further test the proposed factor structure of the 38 items organized in 9 factors specified by the EFA, a CFA with the second sample resulting from the random split was computed (see Table 2). In the CFA, the maximum likelihood estimation method was used and allowed for the factors to correlate among themselves. The results showed an overall good fit for the proposed model, χ^2^(532) = 3938.13, *p* = .00, SRMR = .05, RMSEA = .05, 90% CI [.05, .05], TLI = .91, CFI = .90.

**Table 2.** Results of CFA for the Proposed Factor Structure of the 38 items Organized in 9 Factors and Descriptive Statistics of the Extracted Factors.

	1. Resources	2. Patients in pain	3. Cognitive demands	4. Relationship demands	5. Time demands	6. Physical demands	7. Technical demands	8. Difficult patients	9. Lack of autonomy
Factors									
M	5.94	6.74	8.81	6.44	7.44	5.34	5.67	6.45	4.79
SD	1.93	2.18	1.27	2.51	2.31	2.47	2.42	2.00	2.30
N	4551	4472	4562	4442	4553	4519	4537	4487	4483
AVE	.48	.64	.59	.62	.66	.36	.42	.42	.38
Alpha	.88	.84	.85	.89	.85	.70	.67	.80	.63
Correlations^1^									
1	—	−.03	−.02	−.26	−.23	−.27	−.38	−.21	−.48
2			.18	.23	.18	.12	.16	.54	.13
3				.16	.40	.27	.09	.15	.16
4					.24	.17	.20	.41	.24
5						.29	.29	.24	.37
6							.24	.17	.30
7								.31	.48
8									.29

*Note.* ^1^Correlations are not marked for significance due to the large sample size biasing error estimate and corresponding test statistics.

The results of the localized areas of strain were also in line with the proposed model. First, although approximately one fifth of the standardized residuals were above 2.58, less than 1% were above the 10.00 criteria (*p* < .001). An interpretation of the more extreme values mostly showed items that were within the same factor. Second, approximately one third of the items had modification indexes above 4, but only one fifth were above a more liberal criterion of 10. Akin to the standardized residuals, the highest modification indexes occurred mostly between variables in the same factor and with few exceptions between factors and items from a different factor.

It should be noted that although the more conservative analysis of the standardized residuals and modification indexes suggested potential improvements in the factor model, the more permissive criteria were far more supportive of the model as it had been proposed. Here, it is important to take two aspects into consideration for the use of more permissive criteria. First, the standardized residuals and modification indexes are expected to have more extreme values as the sample sizes are larger. Second, some authors have recommended that models with an overall good fit should be left with no further adjustments to reduce the risk of shaping the model to the idiosyncrasies of the sample error and making it more difficult to replicate in the future (MacCallum et al., 1992). In line with these perspectives, the analysis of the localized areas of strain provided here should be considered more as complementary descriptions of the model than as evidence to further improve the performance of the factor model.

#### Factor Interpretation

In short, the CFA provided further evidence for the 9-factor structure with the 38 items specified by the EFA. The analysis of the data underlying the factor structure revealed that all the factor loadings of the items on the corresponding factors varied (in absolute value) between .48 and .90 (Table S5, Supplementary materials). As with the EFA, for descriptive purposes, the factors were computed using coarse scores (i.e., the average rating of each participant on items composing the factors). The analysis of both the averages and the correlations replicated the results found with the sample used to run the EFA.

#### Alternative Models

Akin to the EFA, the descriptive analysis for the item in the CFA sample reported in the procedure pointed to missing data and data distribution symmetry that could affect the factor model estimation. In order to account for these issues, an MLR with direct ML missing data replacement estimations was computed and compared with the reported ML estimation. The overall model fit with MLR estimation was identical to the model with the ML estimation, χ^2^(532) = 3281.87, *p* = .00, SRMR = .05, RMSEA = .05, 90% CI [.05, .05], TLI = .91, CFI = .90.

## Discussion

Over the past decades, the literature has extensively addressed physicians’ burnout and studied approaches to mitigate its symptoms due to the negative impacts which affect not only physicians but also their families, patients, health care organizations and society (Marques-Pinto et al., 2021; Williams et al., 2020). Against this background, the literature describes physicians’ job characteristics as main predictors of physicians’ burnout symptoms (e.g., Marques-Pinto et al., 2021). However, the research still lacks a systematic description of physicians’ specific job demands and resources, and no consensus regarding the instruments to assess these variables has been found (Aronsson et al., 2017; Lee et al., 2021). Thus, this paper contributes to filling this gap through the design and validation of a theoretically and empirically based measurement instrument of physicians’ job demands and resources.

In line with the JD-R model (Bakker & Demerouti, 2017; Demerouti et al., 2001; Schaufeli & Taris, 2014), the *Physicians’ Job Demands and Resources Scale* is a Portuguese self-report physician-specific job demands and resources measurement instrument, whose dimensions proved to be relevant following an inductive content analysis of the prior literature and an assessment needs validation with physicians. After the identification of relevant dimensions of physician-specific job demands and resources, 44 initial items were generated, and the factor structure was tested with a sample of 9,176 Portuguese Physicians. The results of EFA and CFA with two random split samples provided consistent evidence of a 9-factor structure with 38 of the originally identified 44 items. The 9-factor structure is consistent with the seven dimensions identified in the literature and extend the detail of the *Organizational demands* dimension (into *Technical demands* and *Lack of autonomy*) and of the *Demands of the relationship with patients* dimension (into *Patients in pain* and *Difficult patients*). The alternative models, corrected for data asymmetry and missing data, were also consistent with the 9-factor structure.

Hence, the final version of the *Physicians’ Job Demands and Resources Scale* is composed of nine factors, eight of which cover specific physician-perceived job demands, and one dimension referring to physicians’ perception of job resources. More specifically, the *Resources* scale is related to the perception of positive conditions and opportunities to achieve work goals, stimulate growth and mitigate job demands, at the level of the organization (e.g., “I feel that I have good opportunities for professional development in my workplace.”), interpersonal and social relations (e.g., “I feel that the work climate in my team facilitates my professional activity.”), the organization of work (e.g., “I clearly know what is expected of me in my workplace.”) and/or the task (e.g., “I feel like I get enough feedback on my performance.”). As for the demand-related scales: the *Physical demands* scale refers to the perception of a work environment with adverse physical conditions, risks and hazards (e.g., “I work in an environment where noise levels are very high.”); the *Cognitive demands* scale depicts the perception of a work environment with high levels of mental investment and decision-making requirements, and low error tolerance (e.g., “My job requires me to make many complex decisions.”); the *Technical demands* scale refers to perceived difficulties in acquiring adequate software, diagnostic exams or feedback from other specialties (e.g., “I find it difficult to obtain additional diagnostic tests that I consider necessary.”); the *Lack of autonomy* scale concerns the perception of task-related ambiguity, low autonomy and absent participation in the decision-making processes regarding physicians’ own work (e.g., “I am given incompatible guidelines by different people I work with.”); the *Time demands* refers to excessive work and time requirements (e.g., “I don’t have enough time for the work that needs to be done.”); the *Relationship demands* depicts the perception of interpersonal relations with colleagues and superiors at work marked by interpersonal conflicts, discrimination, harassment, and/or non-recognition of own work (e.g., “If I feel that my work is not recognized.”); the *Difficult patients* scale refers to perceived difficulties in the interpersonal relationship with demanding/problematic patients and their families (e.g., “When I accompany non-cooperative/difficult patients.”); and the *Patients in pain* scale concerns the perceived emotional burden of exposure to patients’ suffering and/or death, and of the responsibility for their health and lives (e.g., “When I accompany terminally ill patients and I am confronted with the death of patients.”).

The findings also supported the adequate internal consistency across the nine factors of the *Physicians’ Job Demands and Resources Scale* (α > .60). The intercorrelations between factors were positive and weak to moderate between the dimensions concerning job demands (except for the two factors regarding the demands of the relationship with patients, the correlation of which was large), and negative and weak to moderate between the job resources factor and the remaining factors. The correlation analysis supported the association of these dimensions, despite being distinct. The analysis of the average explained variance also adds to the psychometric quality of this measure (Lam, 2012; Sinval et al., 2018). Overall, the findings provide support for the psychometric quality of the *Physicians’ Job Demands and Resources Scale* as a self-report measurement instrument to assess physicians’ perceived job characteristics, thus offering an important resource for researchers and practitioners in this field. Since factor analysis suggested a nine-factor solution (instead of a unidimensional score of the 44-items), the *Physicians’ Job Demands and Resources Scale* also provides a more detailed and comprehensive assessment of specific job characteristics perceived by physicians, facilitating the assessment and design of tailored strategies to promote occupational health and prevent physicians’ burnout.

### Limitations and Future Research

Despite its contributions, this study has some limitations that should be addressed. Even though a large sample of physicians participated in the study, a non-probabilistic online sample was used. Also, only Portuguese physicians participated in this research. Therefore, generalization of the findings should be cautious, and further studies are needed to continue to validate the *Physicians’ Job Demands and Resources Scale*’s factor structure and psychometric qualities with different data collection methods, using representative samples, and testing the cross-cultural behavior of the measurement instrument. Further studies including the analysis of additional psychometric qualities, such as test-retest reliability and validity analysis (e.g., criterion-related validity) would also be important contributions to the further assessment and validation of the scale. Future research should also test for structural invariance across various medical specialties which, due to the heterogeneity across the groups, was not tested in this study, as prior literature supports differences regarding the job demands and resources experienced across different medical specialties (e.g., oncologists; Le Blanc & Schaufeli, 2003; Panagioti et al., 2017). The study of structural invariance across gender would also be important since prior literature has pointed to an increase of females in the medical field. As work characteristics differ for males and females (e.g., career opportunities, work-home interference), and some studies have observed different patterns of relationships between job demands/resources and burnout for male and female physicians (Houkes et al., 2008), further research regarding gender differences is recommended. The analysis of the face validity of the items with a higher proportion of missing data (e.g., Physical demands_3 referring to the existence of few resting places for physicians to take a break) suggested these items might not apply as consistently as the remaining items to the work conditions of the physicians in our sample. Nevertheless, the study’s large sample provided the opportunity to analyze the contribution of all the measured working conditions to a factor structure of job resources and demands. Moreover, the *Physicians’ Job Demands and Resources Scale* was developed prior to the Covid-19 pandemic and therefore does not consider the specific demands and resources emerging from this context, which may continue to impact physicians’ job characteristics in the near future. Also, the scale did not integrate a personal resources dimension (a dimension supported by recent literature as an important determinant of physician burnout symptoms; Marques-Pinto et al., 2021) or a scientific-related demands dimension (e.g., constraints related to the pharmaceutical industry and/or ethical requirements). Thus, some adaptations/further developments may be needed and should be investigated. Moreover, the proposed instrument uses a semantic differential response scale anchored with Completely disagree to Completely agree endpoints. These type of scales are common in the literature and, in the context of the present study allow a quicker and accessible response format. This was particularly important as the study population, although highly interested in the study theme, is usually busy. Nevertheless, agree/disagree response formats have important limitations, particularly when compared with item specific responses (e.g., Timbrook et al., 2021). Hence, an important future test to the *Physicians’ Job Demands and Resources Scale* is to adapt the response scale to item specific responses and compare the performance of that version with the original version. This could be accomplished using sub-factor specific measurement scales (e.g., Non-existent resources - Abundant resources, Constant time demands - Rare time demands). Using item specific responses would also allow to, for example, disambiguate if the asymmetry in the responses result from acquiescence or from the prevalence of the phenomena in this population. Lastly, as a self-report measure, the *Physicians’ Job Demands and Resources Scale* is subject to social desirability biases. Although a longer rating scale was used (i.e., 11-point Likert-type response scale; Stöber et al., 2002), future research should consider a measure to test and control the possible effect of social desirability bias.

### Study Impact

Despite the afore-mentioned limitations, by presenting a measurement instrument of physician-specific job demands and resources with good psychometric properties, this study advances important contributions to both research and practice in the field of physician burnout. With the strong associations found between job characteristics and physicians’ burnout (e.g., Marques-Pinto et al., 2021; Panagioti et al., 2017), prior studies have emphasized the need to further comprehend the specific features and impacts of different job characteristics on physicians’ burnout (Chênevert et al., 2021; Lee et al., 2021). Nevertheless, in comparison with other professional groups, the JD-R model has been less studied with physicians, and no robust measure to assess these variables across the various medical specialties and considering the occupation-specific job characteristics has been advanced (Schaufeli & Buunk, 2003; Schaufeli & Taris, 2014). To such end, by presenting a theoretically and empirically grounded and psychometrically validated measurement instrument to assess physicians’ job demands and resources, this study and, specifically, the *Physicians’ Job Demands and Resources Scale,* contributes to the promotion of methodologically robust studies and facilitates advances in the comprehension of physician burnout determinants. Moreover, the validation of the items by independent Portuguese physicians gives additional support to the relevance and adequacy of the dimensions covered within the scale. Also, the use of a large sample provided the opportunity to use the more sophisticated methodological approaches (e.g., to run exploratory and confirmatory factor analyses with two random samples, to test the effect of deleting versus replacing missing data), thus adding to the robustness and reliability of this measure. Lastly, research-wise, with the adequate adaptation of the items’ content, this scale may support the development of different adapted versions for specific groups (e.g., medical specialties), in line with that observed with the Maslach Burnout Inventory (Maslach et al., 1996).

Regarding contributions to practice, this scale may be used to screen and diagnose perceived job demands and resources across specific contexts (e.g., state hospitals) in order for practitioners and managers to promote adequate prevention and intervention policies. We recommend the use of the scale either in the complete format with all the nine subscales or using those subscales relevant to the research questions at hand. We do not recommend the use a single summated measure as this would suggest an underlying construct representing the overall experience of Demands and Resources*,* and assumption that cannot be made without more substantive theoretical considerations.

Prior literature has indicated that organization-targeted interventions addressing job characteristics are the most effective in reducing physician burnout, but also more expensive (Panagioti et al., 2017). Thus, the use of the *Physicians’ Job Demands and Resources Scale* to assess the role and impact of specific job characteristics across work contexts, supports the tailoring of the interventions to the specific needs, thus reducing costs. By being easily administered and scored, the scale also prevents time costs. Finally, since Portuguese is one of the most spoken languages worldwide, and the foremost spoken language in the Southern Hemisphere, the *Physicians’ Job Demands and Resources Scale* can, with the necessary adaptations, ease the expansion of cross-cultural research and practice to prevent physicians’ burnout across health-organizations worldwide. Given the innovation and promising contributions of this measure, with proper translation and adaptation studies, the *Physicians’ Job Demands and Resources Scale* can also present a promising research resource in other sociocultural settings (e.g., an English language version of the measure).

## Supplemental Material

Supplemental Material - Psychometric Assessment of the Physicians’ Job Demands and Resources Scale Click here for additional data file. Supplemental Material for Psychometric Assessment of the Physicians’ Job Demands and Resources Scale by Sérgio Moreira, Sofia Oliveira, Jorge Vala, Rui Costa-Lopes, and Alexandra Marques-Pinto in Evaluation & the Health Professions.

## Data Availability

The datasets presented in this study can be found in online repositories. The names of the repository/repositories and accession number(s) can be found below: Vala, J., Marques-Pinto, A., Moreira, S., Costa Lopes, R., & Januário, P. (2020). Burnout na classe médica em Portugal. *Arquivo Português de Informação Social*, APIS0057.
