# Supplementary material for: Psychometric Assessment of the Physicians’ Job Demands and Resources Scale
Source: Eval Health Prof. 2023 Aug 16;46(4):384–95. doi: 10.1177/01632787231195077 (PMC10637071; doi:10.1177/01632787231195077)
Supplement: Supplemental Material - Psychometric Assessment of the Physicians’ Job Demands and Resources Scale [file sj-pdf-1-ehp-10.1177_01632787231195077.pdf]

Electronic supplementary material

[To accompany Moreira, S., Oliveira, S., Vala, J., Costa-Lopes, R., & Marques-Pinto, A. (2023). Psychometric Assessment of the Physicians' Job Demands and Resources Scale. *Evaluation & the Health Professions*.]

The following supplementary online material includes a full description on the content analysis approach underlying the item generation process of the *Physicians' Job Demands and Resources Scale*, and a detailed report of the results regarding the data analyses computed.

Table S1

Dimensions identified in the literature and corresponding generated items after the inductive content analysis and validation by independent physicians

Job demands and resources identified dimensions (number of items; source)	Name (Lable)	Formulated item (sources)
Physical demands<br>(4 items; Al-Dubai & Rampal, 2010; Bakker et al., 2007; Le Blanc & Schaufeli, 2003; Schaufeli & Taris, 2014)	**Physical demands_1**<br>(Uncomfortable noise)	**“Trabalho num ambiente em que os níveis de ruído são muito elevados.”**<br>Free translation: “I work in an environment where noise levels are very high.”<br>(Suggested by the technical team / focus groups)
	**Physical demands_2**<br>(Uncomfortable temperatures)	**“Trabalho num ambiente demasiado quente / frio.”**<br>Free translation: “I work in a too hot / cold environment.”<br>(Suggested by the technical team / focus groups)
	**Physical demands_3**<br>(Few resting places)	**“No meu local de trabalho há falta de zonas de descanso adequadas para os/as médicos/as em serviço de urgência.”**<br>Free translation: “In my workplace there is a lack of adequate rest areas for doctors on call.”<br>(*Lack of comfortable rooms for doctors on call*, Al-Dubai & Rampal, 2010).
	**Physical demands_4**<br>(High exposure to risk)	**“No meu trabalho estou muito exposto a riscos físicos (e.g., instrumentos cortantes; químicos tóxicos; sangue contaminado com agentes patogénicos).”**

		Free translation: "At my work I am very exposed to physical hazards (e.g., sharp instruments, toxic chemicals, blood contaminated with pathogens)."<br>(*To apply toxic treatments*, Le Blanc & Schaufeli, 2003; *Ergonomics and work-related hazards*, Lee et al, 2013; *Risks and hazards*, Schaufeli & Taris, 2014).
Cognitive demands<br>(4 items; Le Blanc & Schaufeli, 2003; Schaufeli et al., 2009; Schaufeli & Taris, 2014)	**Cognitive demands_1**<br>(High level of concentration)	**"O meu trabalho exige-me níveis de atenção / concentração / precisão muito elevados."**<br>Free translation: "My work requires very high levels of attention / concentration / precision."<br>(*Does your work demand a lot of concentration?*, Schaufeli et al, 2009)
	**Cognitive demands_2**<br>(Multitasking)	**"O meu trabalho exige que me lembre de muitas informações simultaneamente."**<br>Free translation: "My job requires me to remember a lot of information at once."<br>(*Amount of knowledge*, Le Blanc & Schaufeli, 2003)
	**Cognitive demands_3**<br>(Complex decisions)	**"O meu trabalho exige-me que tome muitas decisões complexas."**<br>Free translation: "My job requires me to make many complex decisions."<br>(*Decision making has become more complicated*, Le Blanc & Schaufeli, 2003)
	**Cognitive demands_4**<br>(Decisions with dreadful consequences)	**"Se cometer erros no meu trabalho as consequências para os doentes são muito graves."**<br>Free translation: "If I make mistakes in my work, the consequences for the patients are very serious."<br>(Suggested by the technical team / focus groups)

Organizational demands (10 items; Maslach et al., 2001; Schaufeli et al., 2009, 2011; Schaufeli & Taris, 2014)	**Organizational demands_1** (Excessive number of meetings)	**“Sou convocado/a para um número excessivo de reuniões.”** Free translation: “I am required to attend an excessive number of meetings.” (*Do you have a lot of meetings*?, Schaufeli et al, 2009)
	**Organizational demands_2** (Lack of other physicians)	**“A falta de médicos/as no meu local de trabalho dificulta a minha prática.”** Free translation: “The lack of physicians at my workplace makes my practice difficult.” (Sho*rtage of physicians makes my practice more difficult*, Lee et al., 2008; *difficulty in finding a locum*, Al-Dubai & Rampal, 2010)
	**Organizational demands_3** (Excessive bureaucracy)	**“A quantidade de trabalho burocrático que tenho é excessiva.”** Free translation: “The amount of paperwork I have is excessive.” (*Bureaucracy*, Le Blanc & Schaufeli, 2003; *the amount of paperwork to do is far too much*, Lee at al., 2008; *paperwork*, Schaufeli et al., 2011; *paperwork volume*, Tokuda et al., 2009)
	**Organizational demands_4** (Lack of autonomy)	**“No meu trabalho sinto que há demasiadas imposições e tenho falta de autonomia no que faço.”** Free translation: “In my work I feel that there are too many impositions and I lack autonomy in what I do.” (*Lack of autonomy*, Maslach et al., 2001; *decreased professional autonomy*, Schaufeli et al., 2011).
	**Organizational**	**“Sinto que não me é dada a possibilidade de participar na tomada de**

	**demands_5**<br>(Lack of participation)	**decisões que afetam o meu trabalho.”**<br>Free translation: “I feel that I am not given the opportunity to participate in decision making that affect my work.”<br>(*Little participation in decision making*, Maslach et al., 2001)
	**Organizational demands_6**<br>(Difficulties in obtaining exams)	**“Sinto dificuldade em obter exames complementares de diagnóstico que considero necessários.”**<br>Free translation: “I find it difficult to obtain additional diagnostic tests that I consider necessary.”<br>(*Long waits for accessing diagnostic tests*, Lee et al., 2008; *diagnostic tests*, Tokuda et al., 2009).
	**Organizational demands_7**<br>(Difficulties in having feedback from other areas)	**“Sinto dificuldade em obter o feedback necessário dos especialistas de outras áreas.”**<br>Free translation: “I find it difficult to get the necessary feedback from experts in other areas.”<br>(*Long waits for accessing specialists*, Lee et al., 2008)
	**Organizational demands_8**<br>(Inadequate software)	**“No meu trabalho os recursos informáticos / sistema informático são inadequados.”**<br>Free translation: “In my work, the informatic resources / software are inadequate.”<br>(*Computer problems,* Schaufeli & Taris, 2014)
	**Organizational**	**“São-me dadas orientações incompatíveis entre si por diferentes pessoas**

	**demands_9** (Incompatible instructions)	**com quem trabalho.”** Free translation: “I am given incompatible guidelines by different people I work with.” (*Role conflict: Conflicting demands at the job have to be met*, Lee et al, 2013; Maslach et al., 2001, Schaufeli & Buunk, 2003, Schaufeli & Taris, 2014)
	**Organizational demands_10** (Excessive time with technical instruments)	“**No meu trabalho passo muito tempo a lidar com equipamentos técnicos**.” Free translation: “In my work I spend a lot of time dealing with technical equipment.” (*High tech demands*, *technical demands*, Le Blanc & Schaufeli, 2003)
Demands of the relation with patients (8 items; Al-Dubai & Rampal, 2010; Bakker et al., 2000, 2007; Le Blanc & Schaufeli, 2003; Lee et al., 2013; Maslach et al., 2001; Schaufeli & Buunk, 2003; Schaufeli & Taris, 2014; Schaufeli et al., 2011)	**Demands of the relation with patients_1** (Suffering patients)	“**Quando me relaciono com doentes em sofrimento.**” Free translation: “When I relate with suffering patients.” (*Repeated exposure to suffering*, Le Blanc & Schaufeli, 2003; *patient suffering and emotions*, Lee et al., 2013; *confrontation with suffering patients*, Schaufeli et al., 2011).
	**Demands of the relation with patients_2** (Terminal patients)	**“Quando acompanho doentes em processo terminal e me confronto com a morte de doentes.”** Free translation: “When I accompany terminally ill patients and I am confronted with the death of patients.” (*Contact with chronically or terminally ill patients, confrontation with death and dying*, Maslach et al., 2001, Schaufeli & Buunk, 2003; *frequent exposure to death*, Le Blanc & Schaufeli, 2003).

	**Demands of the relation with patients_3** (Responsibility over patients' lives)	"**Se sinto responsabilidade sobre a saúde e a vida dos doentes**." Free translation: "If I feel responsible for the health and lives of patients." (O*verwhelmed by the level of responsibility*, Le Blanc & Schaufeli, 2003, Maslach et al., 2001; *sense of professional obligation*, Lee et al., 2008).
	**Demands of the relation with patients_4** (Non-cooperative patients)	**"Quando acompanho doentes não colaborantes / difíceis."** Free translation: "When I accompany non-cooperative / difficult patients." (*Uncooperative patients*, LeBlanc & Schaufeli, 2003; *interaction with difficult patients*, Schaufeli & Buunk, 2003; *dealing with problem patients*, Al-Dubai & Rampal, 2010, Schaufeli et al., 2011; *confrontation with demanding patients*, Schaufeli et al., 2011; *a patient who insisted on referral to a consultant although you did not regard the referral as necessary*, Bakker et al., 2000, Schaufeli et al., 2011)
	**Demands of the relation with patients_5** (Threatening patients)	**"Se lido com doentes que me ameaçam."** Free translation: "If I deal with patients who threaten me." (*A patient who threatened to write to the disicplinary commitee to complain about you, a patient who threatens you physically*, Bakker et al., 2000, Schaufeli et al., 2011; *patient harassment*, Bakker et al., 2007; *incivility*, Lee et al, 2013; *harassment by patients*, Schaufeli & Taris, 2014; *patient aggression*, Schaufeli et al., 2011)
	**Demands of the relation with patients_6**	**"Se lido com doentes com problemas que estão fora da minha área de intervenção."**

	(Patients with unfamiliar conditions)	Free translation: "If I deal with patients with problems that are outside my area of intervention."<br>(*Dealing with patient's psychosocial problems*, Al-Dubai & Rampal, 2010)
	**Demands of the relation with patients_7**<br>(Patients' family)	**"Quando me relaciono com os familiares dos doentes."**<br>Free translation: "When I relate with the patients' relatives."<br>(*Family desperation, to satisfy the emotional needs of families*, Le Blanc & Schaufeli, 2003)
	**Demands of the relation with patients_8**<br>(Patients lack of recognition)	**"Se sinto falta de reconhecimento dos doentes pelo meu trabalho."**<br>Free translation: "If I feel lack of recognition from patients for my work."<br>(*Lack of appreciation from patients*, Al-Dubai & Rampal, 2010; *meet with little appreciation*, Bakker et al., 2000)
Relations in the workplace demands (5 items; Al-Dubai & Rampal, 2010; Le Blanc & Schaufeli, 2003; Lee et al., 2013; Maslach et al., 2001; Schaufeli & Taris, 2014)	**Relations in the workplace demands_1**<br>(Conflict situations)	**"Se experiencio situações de conflito."**<br>Free translation: "If I experience conflict situations."<br>(*Negative aspects of relation with colleagues: conflicts*, Le Blanc & Schaufeli, 2003; *conflicts*, Lee et al, 2013; *unresolved conflict with others on the job*, Maslach et al., 2001; *interpersonal conflict*, Schaufeli & Taris, 2014)
	**Relations in the workplace demands_2**<br>(Colleagues lack of recognition)	**"Se sinto que o meu trabalho não é reconhecido."**<br>Free translation: "If I feel that my work is not recognized."<br>(Ne*gative aspects of relation with colleagues: lack of appreciation*, Le Blanc & Schaufeli, 2003; *poor status in the eyes of your seniors*, Al-Dubai & Rampal, 2010; *feeling undervalued*, Lee et al., 2008)

	**Relations in the workplace demands_3** (Negative discrimination)	**“Se me sinto negativamente discriminado/a.”** Free translation: “If I feel negatively discriminated against.” (Suggested by the technical team / focus groups)
	**Relations in the workplace demands_4** (Moral bullying)	**“Se me sinto vítima de assédio moral.”** Free translation: “If I feel like a victim of moral harassment.” (Suggested by the technical team / focus groups)
	**Relations in the workplace demands_5** (Questioned for my performance)	**“Se sinto que o meu trabalho é posto em causa.”** Free translation: “If I feel that my work is being called into question.” (*Little sympathy for the other’s viewpoint*, Le Blanc & Schaufeli, 2003)
Quantitative demands relative to workload / time pressure (4 items; LeBlanc & Schaufeli, 2003; Maslach et al., 2001; Montgomery et al., 2006; Schaufeli & Bakker, 2004; Schaufeli & Buunk, 2003)	**Time demands_1** (Permeant availability)	**“Sinto que tenho de estar disponível como médico/a a qualquer hora.”** Free translation: “I feel like I have to be available as a doctor at all times.” (*Time pressure*, Le Blanc & Schaufeli, 2003, Maslach et al., 2001, Schaufeli & Bakker, 2004, Schaufeli & Buunk, 2003, Schaufeli & Taris, 2014)
	**Time demands_2** (Excessive number of hours of work)	**“Sinto que trabalho um número de horas excessivo.”** Free translation: “I feel like I work an excessive number of hours.” (*Long work hours*, Al-Dubai & Rampal, 2010; *work hours*, Tokuda et al., 2009).
	**Time demands_3** (Extensive time investment)	**“O meu trabalho exige-me que trabalhe arduamente.”** Free translation: “My job requires me to work hard.” (*My job requires working very hard*, Schaufeli & Bakker, 2004; *increased workload*, Al-Dubai & Rampal, 2010; *workload*, Houkes et al., 2008, Lee et al,

		2013, Maslach et al., 2001, Schaufeli & Buunk, 2003; *work overload*, Montgomery et al., 2006, Schaufeli & Taris, 2014; *work volume*, Tokuda et al., 2009)
	**Time demands_4**<br>(Lack of time to perform the work needed)	**“Não tenho tempo suficiente para o trabalho que há a fazer.”**<br>Free translation: “I don't have enough time for the work that needs to be done.”<br>(*Too much work to do in too little time*, Montgomery et al., 2006)
Job resources<br>(9 items; Bakker et al., 2007; LeBlanc & Schaufeli, 2003; Schaufeli & Bakker, 2004; Schaufeli & Taris, 2014)	**Job resources_1**<br>(Clear work expectancies)	**“Sei claramente o que se espera de mim no meu local de trabalho.”**<br>Free translation: “I clearly know what is expected of me in my workplace.”<br>(Org*anization of the work: role clarity*, Bakker et al., 2007; *goal clarity*, Schaufeli & Taris, 2014)
	**Job resources_2**<br>(Participations in important decisions)	**“Sinto que me é dada a possibilidade de participar na tomada de decisões que afetam o meu trabalho.”**<br>Free translation: “I feel that I am given the opportunity to participate in decision making that affect my work.”<br>(*Organization of the work: participation in decision making*, Bakker et al., 2007, Schaufeli & Taris, 2014; *decision involvement*, Schaufeli & Bakker, 2004)
	**Job resources_3**<br>(Secure work conditions)	**“Sinto que o meu local de trabalho está organizado de acordo com políticas, procedimentos e práticas de segurança no trabalho adequados.”**<br>Free translation: “I feel that my workplace is organized according to proper workplace safety policies, procedures and practices.”<br>(*Organization: safety climate*, Schaufeli & Taris, 2014)

	**Job resources_4** (Job progression opportunities)	**"Sinto que tenho boas oportunidades de desenvolvimento profissional no meu local de trabalho."** Free translation: "I feel that I have good opportunities for professional development in my workplace." (*Organization: opportunities for professional development*, Schaufeli & Taris, 2014; *career opportunities*, Bakker et al., 2007; *developmental / career prospects*, LeBlanc & Schaufeli, 2003; *professional development*, Lee et al., 2013)
	**Job resources_5** (Good teamwork environment)	**"Sinto que o clima de trabalho na minha equipa facilita a minha atividade profissional."** Free translation: "I feel that the work climate in my team facilitates my professional activity." (*Interpersonal and social relations: The team can be an important resource*, Le Blanc & Schaufeli, 2003; *team cohesion*, Schaufeli & Taris, 2014)
	**Job resources_6** (Good technical resources)	**"Os recursos técnicos e equipamentos de que disponho no local de trabalho facilitam a minha atividade profissional."** Free translation: "The technical resources and equipment I have at my workplace make my professional activity easier." (Suggested by the technical team / focus groups).
	**Job resources_7** (Feedback about my	**"Sinto que recebo feedback suficiente sobre o meu desempenho."** Free translation: "I feel like I get enough feedback on my performance."

	performance)	(*Task: performance feedback*, Bakker et al., 2007, Schaufeli & Bakker, 2004, Schaufeli & Taris, 2014; *feedback*, Lee et al., 2013).
	**Job resources_8**<br>(Autonomy with my work)	**"Sinto que tenho um bom nível de autonomia no trabalho que faço."**<br>Free translation: "I feel I have a good level of autonomy in the work I do."<br>(*Task: autonomy*, Bakker et al., 2007, LeBlanc & Schaufeli, 2003, Lee et al., 2013, Schaufeli & Bakker, 2004).
	**Job resources_9**<br>(Available help)	**"Sinto que posso contar com ajuda no meu local de trabalho."**<br>Free translation: "I feel like I can count on help in my workplace."<br>(*Interpersonal and social relations: supervisor and coworker support, social support*, Bakker et al., 2007; *support from colleagues and supervisors*, Houkes et al., 2008; *peer support*, Lee et al., 2013; *support from colleagues*, Schaufeli & Bakker, 2004; *social support from colleagues and social support from supervisor*, Schaufeli & Taris, 2014)

Table S2

Items included in the final version of the scale (full list with excluded items available upon request to the authors)

Item	Name	Label	M	SD	Skewness	Kurtosis	N
**1**	Physical demands_1	Uncomfortable noise	4.86	3.06	0.78	-15.18	8830
**2**	Physical demands_2	Uncomfortable temperatures	4.93	3.14	-0.11	-15.41	8815
**3**	Physical demands_3	Few resting places	6.58	3.36	-15.46	-11.52	7169
**4**	Physical demands_4	High exposure to risk	5.49	3.41	-3.71	-17.67	8419
**5**	Cognitive demands_1	High level of concentration	8.93	1.37	-46.25	57.26	9115
**6**	Cognitive demands_2	Multitasking	8.99	1.31	-49.08	67.75	9111
**7**	Cognitive demands_3	Complex decisions	8.69	1.55	-39.39	36.33	9106
**8**	Cognitive demands_4	Decisions with dreadful consequences	8.51	1.87	-40.89	30.48	9107
**9**	Organizational demands_1	Excessive number of meetings	4.94	2.94	-1.27	-13.00	8672
**10**	Organizational demands_2	Lack of other physicians	6.64	3.20	-18.62	-9.81	8573
**11**	Organizational demands_3	Excessive bureaucracy	7.70	2.60	-31.03	7.69	8858
**12**	Organizational demands_4	Lack of autonomy	5.39	2.94	-3.97	-13.28	8849
**13**	Organizational demands_5	Lack of participation	5.52	3.03	-4.54	-14.36	8855
**14**	Organizational demands_6	Difficulties in obtaining exams	4.58	3.19	4.27	-16.35	8554
**15**	Organizational demands_7	Difficulties in having feedback from other areas	5.77	2.91	-9.00	-12.63	8891
**16**	Organizational demands_8	Inadequate software	6.52	3.15	-15.59	-12.45	9000
**17**	Organizational demands_9	Incompatible instructions	4.12	3.03	7.76	-13.71	8553
**18**	Organizational demands_10	Excessive time with technical instruments	6.27	3.09	-14.16	-11.58	8680
**19**	Demands of the relation with patients_1	Suffering patients	6.39	2.46	-17.43	-1.11	8811
**20**	Demands of the relation with patients_2	Terminal patients	6.68	2.61	-17.97	-3.32	8036
**21**	Demands of the relation with patients_3	Responsibility over patients' lives	7.12	2.44	-24.46	4.70	8859
**22**	Demands of the relation with patients_4	Non-cooperative patients	7.03	2.33	-21.73	3.22	8686

Item	Name	Label	M	SD	Skewness	Kurtosis	N
**23**	Demands of the relation with patients_5	Threatening patients	7.00	2.84	-20.41	-4.21	7877
**24**	Demands of the relation with patients_6	Patients with unfamiliar conditions	6.77	2.47	-17.26	-1.60	8443
**25**	Demands of the relation with patients_7	Patients' family	5.82	2.55	-10.57	-5.54	8727
**26**	Demands of the relation with patients_8	Patients lack of recognition	5.86	2.84	-8.38	-10.93	8735
**27**	Relations in the workplace demands_1	Conflict situations	7.19	2.55	-25.11	3.20	8689
**28**	Relations in the workplace demands_2	Colleagues lack of recognition	6.93	2.66	-22.11	-1.39	8695
**29**	Relations in the workplace demands_3	Negative discrimination	6.14	3.23	-18.55	-7.43	8183
**30**	Relations in the workplace demands_4	Moral bullying	4.67	3.71	-12.84	-13.14	7334
**31**	Relations in the workplace demands_5	Questioned for my performance	6.61	2.93	2.15	-18.54	8482
**32**	Job resources_1	Clear work expectancies	7.22	2.31	-25.75	7.12	9058
**33**	Job resources_2	Participations in important decisions	5.51	2.77	-9.97	-10.37	8945
**34**	Job resources_3	Secure work conditions	5.46	2.79	-8.83	-11.14	9017
**35**	Job resources_4	Job progression opportunities	5.01	2.79	-5.92	-12.77	8960
**36**	Job resources_5	Good teamwork environment	6.15	2.83	-16.14	-7.22	8882
**37**	Job resources_6	Good technical resources	5.31	2.77	-7.43	-11.49	8990
**38**	Job resources_7	Feedback about my performance	4.57	2.65	-0.33	-11.86	9002
**39**	Job resources_8	Autonomy with my work	6.82	2.26	-24.03	7.19	9030
**40**	Job resources_9	Available help	6.61	2.50	-20.49	-0.04	8997
**41**	Time demands_1	Permeant availability	6.17	3.01	-16.08	-9.51	9067
**42**	Time demands_2	Excessive number of hours of work	7.45	2.79	-30.08	4.42	9076
**43**	Time demands_3	Extensive time investment	7.81	2.28	-37.06	23.07	9048
**44**	Time demands_4	Lack of time to perform the work needed	6.92	2.83	-22.17	-3.71	9075

Table S3

Detailed results of the factorial structure of the EFA and descriptive statistics of the extracted factors regarding the third factorial model

	1. Resources	2. Patients in pain	3. Cognitive demands	4. Relationship demands	5. Time demands	6. Physical demands	7. Technical demands	8. Difficult patients	9. Lack of autonomy
**Items**									
Job resources_4	**-0.82**	-0.02	0.02	-0.01	-0.04	-0.04	-0.07	0.02	0.18
Job resources_2	**-0.74**	0.00	0.03	0.02	0.01	0.03	0.02	0.03	-0.06
Job resources_7	**-0.72**	-0.02	0.03	0.02	-0.06	-0.04	-0.02	-0.01	0.09
Job resources_9	**-0.71**	0.00	0.00	0.04	0.00	0.07	-0.01	-0.01	-0.07
Job resources_5	**-0.70**	0.02	-0.01	0.07	-0.03	-0.01	0.05	-0.02	-0.02
Job resources_3	**-0.63**	0.03	0.01	-0.05	-0.06	-0.17	-0.14	-0.01	0.13
Job resources_8	**-0.51**	0.01	-0.01	0.04	0.06	0.07	-0.08	0.03	-0.36
Job resources_1	**-0.42**	0.00	-0.11	-0.02	0.02	-0.03	0.04	0.04	-0.20
Demands of the relation with patients_1	-0.02	**0.92**	0.01	-0.05	0.01	0.02	0.05	0.06	-0.02
Demands of the relation with patients_2	0.00	**0.85**	0.02	-0.05	-0.01	0.00	-0.01	0.00	0.00
Demands of the relation with patients_3	0.03	**0.51**	-0.10	0.05	0.02	-0.01	-0.02	-0.28	0.02
Cognitive demands_2	-0.01	-0.02	**-0.85**	0.00	0.01	-0.05	0.06	-0.07	0.01
Cognitive demands_1	0.01	0.02	**-0.84**	-0.01	-0.01	0.00	0.00	0.02	-0.03
Cognitive demands_3	-0.01	-0.05	**-0.83**	0.01	0.05	0.01	0.03	-0.02	0.05
Cognitive demands_4	0.01	0.06	**-0.64**	-0.02	0.02	0.08	-0.06	0.04	0.01
Relations in the workplace demands_3	0.01	-0.01	0.00	**-0.91**	-0.01	0.00	-0.01	0.03	0.01
Relations in the workplace demands_5	-0.03	0.01	0.00	**-0.89**	0.00	0.00	0.00	-0.01	0.03

	1. Resources	2. Patients in pain	3. Cognitive demands	4. Relationship demands	5. Time demands	6. Physical demands	7. Technical demands	8. Difficult patients	9. Lack of autonomy
Relations in the workplace demands_4	-0.03	-0.01	0.00	**-0.78**	-0.02	-0.01	0.05	0.06	-0.01
Relations in the workplace demands_2	0.10	0.02	-0.02	**-0.71**	0.05	0.02	-0.02	-0.13	0.01
Relations in the workplace demands_1	0.03	0.10	0.00	**-0.63**	0.05	0.04	-0.03	-0.11	-0.02
Time demands_2	0.04	0.01	0.04	-0.02	**0.83**	0.05	-0.05	0.01	-0.02
Time demands_3	-0.01	0.00	-0.15	-0.01	**0.83**	-0.03	-0.02	0.03	-0.04
Time demands_4	0.05	0.00	0.02	-0.01	**0.75**	-0.05	0.07	-0.06	0.08
Physical demands_1	-0.01	0.01	0.05	0.00	0.04	**0.74**	-0.03	-0.02	0.00
Physical demands_2	-0.03	0.02	0.08	-0.03	0.00	**0.69**	0.08	-0.02	-0.03
Physical demands_4	0.00	0.00	-0.18	-0.04	-0.02	**0.52**	-0.09	0.02	0.03
Physical demands_3	0.09	-0.01	-0.06	0.01	0.01	**0.42**	0.07	-0.03	0.03
Organizational demands_7	0.03	0.03	-0.01	-0.01	0.00	-0.07	**0.79**	-0.07	-0.06
Organizational demands_6	-0.03	0.01	-0.01	-0.02	-0.01	0.02	**0.70**	0.02	0.06
Organizational demands_8	0.07	0.00	-0.02	0.04	0.09	0.19	**0.37**	-0.02	0.08
Demands of the relation with patients_6	0.00	0.03	-0.06	-0.01	-0.01	-0.01	0.06	**-0.68**	-0.02
Demands of the relation with patients_7	0.00	0.11	0.05	0.09	0.00	0.07	0.00	**-0.65**	0.08
Demands of the relation with patients_5	-0.05	-0.08	-0.03	-0.15	0.01	-0.01	0.02	**-0.64**	-0.06
Demands of the relation with patients_4	0.01	0.25	-0.06	0.07	0.02	-0.06	-0.01	**-0.58**	0.00
Demands of the relation with patients_8	0.06	-0.05	0.03	-0.10	0.04	0.05	0.02	**-0.55**	0.01
Organizational demands_4	0.22	0.04	-0.05	-0.09	0.03	0.03	0.18	-0.04	**0.45**

	1. Resources	2. Patients in pain	3. Cognitive demands	4. Relationship demands	5. Time demands	6. Physical demands	7. Technical demands	8. Difficult patients	9. Lack of autonomy
Organizational demands_1	-0.10	0.01	-0.03	-0.01	0.21	0.06	0.06	-0.02	**0.36**
Organizational demands_9	0.22	0.00	0.00	-0.08	-0.03	0.16	0.25	-0.04	**0.34**
**Factors**									
Var. (%)	20.78	9.93	7.16	5.84	3.24	2.91	2.58	1.79	1.28
Alpha / r	0.88	0.84	0.87	0.90	0.86	0.86	0.69	0.80	0.61
M	5.93	6.72	8.75	6.37	7.34	5.31	5.62	6.46	4.82
SD	1.95	2.20	1.31	2.59	2.34	2.46	2.43	1.97	2.28
N	4540	4477	4556	4423	4536	4508	4533	4472	4474
**Correlations**[1]									
1	-	-0.06	0.00	-0.26	-0.23	-0.29	-0.39	-0.19	-0.48
2			0.20	0.22	0.16	0.11	0.15	0.54	0.18
3				0.18	0.39	0.27	0.11	0.19	0.13
4					0.23	0.18	0.17	0.38	0.27
5						0.28	0.28	0.25	0.36
6							0.26	0.18	0.31
7								0.29	0.48
8									0.31

Note. [1]Correlations are not marked for significance due the large sample size biasing error estimate and corresponding test statistics.

Table S4

List of items and corresponding factors in the EFA analysis with the original data, transformed data, and data using the listwise deletion

Item	Full list of items	Original data	Transformed data	Listwise deletion
**1**	Physical demands_1	pD	pD	pD
**2**	Physical demands_2	pD	pD	pD
**3**	Physical demands_3	pD	pD	pD
**4**	Physical demands_4	pD	pD	pD
**5**	Cognitive demands_1	cD	cD	cD
**6**	Cognitive demands_2	cD	cD	cD
**7**	Cognitive demands_3	cD	cD	cD
**8**	Cognitive demands_4	cD	cD	cD
**9**	Organizational demands_1	Dla	cross loading	Dg
**10**	Organizational demands_2	low loadings	low loadings	low loadings
**11**	Organizational demands_3	cross loading	cross loading	cross loading
**12**	Organizational demands_4	Dla	cross loading	Dg
**13**	Organizational demands_5	cross loading	cross loading	cross loading
**14**	Organizational demands_6	Dltr	Dg	Dg
**15**	Organizational demands_7	Dltr	Dg	Dg
**16**	Organizational demands_8	Dltr	Dg	Dg
**17**	Organizational demands_9	Dla	Dg	low loadings
**18**	Organizational demands_10	low loadings	low loadings	low loadings
**19**	Demands of the relation with patients_1	Drps	Drps	Drps
**20**	Demands of the relation with patients_2	Drps	Drps	Drps
**21**	Demands of the relation with patients_3	Drps	Drps	Drps
**22**	Demands of the relation with patients_4	Drdp	Drdp	Drdp
**23**	Demands of the relation with patients_5	Drdp	Drdp	Drdp

Item	Full list of items	Original data	Transformed data	Listwise deletion
**24**	Demands of the relation with patients_6	Drdp	Drdp	Drdp
**25**	Demands of the relation with patients_7	Drdp	Drdp	Drdp
**26**	Demands of the relation with patients_8	Drdp	Drdp	Drdp
**27**	Relations in the workplace demands_1	rwD	rwD	rwD
**28**	Relations in the workplace demands_2	rwD	rwD	rwD
**29**	Relations in the workplace demands_3	rwD	rwD	rwD
**30**	Relations in the workplace demands_4	rwD	rwD	rwD
**31**	Relations in the workplace demands_5	rwD	rwD	rwD
**32**	Job resources_1	jR	jR	jR
**33**	Job resources_2	jR	jR	jR
**34**	Job resources_3	jR	jR	jR
**35**	Job resources_4	jR	jR	jR
**36**	Job resources_5	jR	jR	jR
**37**	Job resources_6	cross loading	cross loading	cross loading
**38**	Job resources_7	jR	jR	jR
**39**	Job resources_8	jR	jR	jR
**40**	Job resources_9	jR	jR	jR
**41**	Time demands_1	low loadings	tD	tD
**42**	Time demands_2	tD	tD	tD
**43**	Time demands_3	tD	tD	tD
**44**	Time demands_4	tD	tD	tD

Notes. pD = Physical demands; cD = Cognitive demands; Dla = Demands due to the lack of autonomy / participation in decision making; Dg = Demands in general; Dltr = Demands due to the lack of adequate technical and diagnostic resources; Drps = Demands of the relation with patients in suffering; Drdp = Demands of the relation with difficult patients; rwD = Relations in the workplace demands; jR = Job resources; tD = Quantitative demands relative to workload / time pressure.

The factor *Demands in general* (Dg) is only presented in this table to provide a simple name to the single factor resulting from the analyses with transformed data and listwise deletion.

Table S5

CFA results regarding the factor loadings of the items on the corresponding factors

	Resources	Patients in suffering	Cognitive demands	Relationship demands	Time demands	Physical demands	Technical demands	Difficult patients	Lack of autonomy
Job resources_4	0.78								
Job resources_2	0.72								
Job resources_7	0.71								
Job resources_9	0.72								
Job resources_5	0.71								
Job resources_3	0.70								
Job resources_8	0.63								
Job resources_1	0.50								
Demands of the relation with patients_1		0.90							
Demands of the relation with patients_2		0.88							
Demands of the relation with patients_3		0.66							
Cognitive demands_2			0.87						
Cognitive demands_1			0.84						
Cognitive demands_3			0.84						
Cognitive demands_4			0.61						
Relations in the workplace demands_3				0.86					
Relations in the workplace demands_5				0.89					
Relations in the workplace demands_4				0.71					

	Resources	Patients in suffering	Cognitive demands	Relationship demands	Time demands	Physical demands	Technical demands	Difficult patients	Lack of autonomy
Relations in the workplace demands_2				0.78					
Relations in the workplace demands_1				0.74					
Time demands_2					0.82				
Time demands_3					0.89				
Time demands_4					0.77				
Physical demands_1						0.71			
Physical demands_2						0.71			
Physical demands_4						0.52			
Physical demands_3						0.48			
Organizational demands_7							0.74		
Organizational demands_6							0.72		
Organizational demands_8							0.49		
Demands of the relation with patients_6								0.69	
Demands of the relation with patients_7								0.69	
Demands of the relation with patients_5								0.67	
Demands of the relation with patients_4								0.72	
Demands of the relation with patients_8								0.59	
Organizational demands_4									0.70
Organizational demands_1									0.36
Organizational demands_9									0.73
